# External validity and anchoring heuristics: application of DUNDRUM-1 to secure service gatekeeping in South Wales

**DOI:** 10.1192/bjb.2017.6

**Published:** 2018-02

**Authors:** Daniel Lawrence, Tracey-Lee Davies, Ruth Bagshaw, Paul Hewlett, Pamela Taylor, Andrew Watt

**Affiliations:** 1 Priory Group & Partnerships in Care, UK; 2 School of Health Sciences, Cardiff Metropolitan University; 3 South Wales Forensic Mental Health Service, Bridgend; 4 Division of Psychological Medicine, School of Medicine, Cardiff University

## Abstract

**Aims and method:**

Structured clinical judgement tools provide scope for the standardisation of forensic service gatekeeping and also allow identification of heuristics in this decision process. The DUNDRUM-1 triage tool was completed retrospectively for 121 first-time referrals to forensic services in South Wales. Fifty were admitted to medium security, 49 to low security and 22 remained in open conditions.

**Results:**

DUNDRUM-1 total scores differed appropriately between different levels of security. However, regression revealed heuristic anchoring on the ‘legal process’ and ‘immediacy of risk due to mental disorder’ items.

**Clinical implications:**

Patient placement was broadly aligned with DUNDRUM-1 recommendations. However, not all triage items informed gatekeeping decisions. It remains to be seen whether decisions anchored in this way are effective.

**Declaration of interest:**

Dr Mark Freestone gave permission for AUC values from Freestone *et al*. (2015) to be presented here for comparison.

Secure mental health services in the United Kingdom are designed to detain individuals who have severe mental ill health and are believed to pose a significant risk of harm to others.^1^ Secure services in the UK are configured around three tiers (low, medium and high) in order to accommodate people in different levels of clinical security. The task of allocating individual patients to different security tiers is challenging. First, clinicians are expected to adhere to the principle of the *least restrictive option*,^2^ and yet there are no clearly defined eligibility criteria for different levels of security, and no clear guidelines for their particular roles.^3^ Furthermore, consideration of a patient's clinical presentation, psychiatric history, diagnosis, comorbidity, developmental history, family history, employment history and ethnic/cultural background,^4^ and the patchy information about outcomes achieved by secure services, complicates and compromises gatekeeping decisions further still. The challenge for forensic gatekeepers in matching patients to appropriate security levels is apparent in the reported heterogeneity of cases admitted by both medium^5^^,^^6^ and low^7^ secure services.

The process of deciding who should/should not be admitted to specific levels of security requires consideration of a wide range of characteristics.^4^ Unfortunately, knowledge about associations between patient characteristics and relevant outcomes is currently restricted by a dearth of rigorously controlled outcome studies^8^ and by a range of confounding factors that include, but are not restricted to, diagnostic uncertainty, individualistic risks, individual needs and treatment responsivity. At present, decisions regarding patient allocation to different levels of security must be extrapolated from an uncertain knowledge base, with inevitable uncertainty regarding likely outcomes. Apart from the weak knowledge base, it is important also to consider the inherent complexity of this form of decision process.

Under complex and uncertain circumstances, human decision-making typically depends upon heuristics. Heuristics describe the normal tendency for people to employ a range of strategies in order to reduce the burden on their finite cognitive resources.^9^ Heuristics are especially prevalent when decision makers are required to accommodate multiple or conflicting sources of information.^10^

Heuristic phenomena of particular relevance to forensic practice include representativeness bias, availability heuristics and decision anchoring.^11^ The anchoring heuristic, which will be the focus here, describes decision processes that are biased toward initially received (or attended to) information.^12^^,^^13^ Anchoring describes a process where initially considered features disproportionately influence decisions, while later processed information is neglected and does not influence decision-making.^10^

When anchoring is present, decisions that are supposed to be based on a range of characteristics (i.e. should the patient be admitted to secure services?) will instead be based on a limited set of characteristics. Under these circumstances, the structure of assessments and/or the emphasis placed on specific characteristics will be more important than the bulk of the information that is intended to inform decisions. There is a risk, therefore, that patients may be inappropriately placed because of the neglect of information.

One of the objectives of structured clinical judgement tools is to increase the transparency and consistency in decision-making and to make sure that relevant factors consistently inform clinical decisions. Despite the availability and widespread knowledge of structured clinical judgement tools in forensic services, clinical opinion can still be more influential than formally assessed risk status in decisions to detain people in secure conditions.^14^

Clinical opinion has been criticised as error prone and too heavily dependent on heuristics.^15^ Heuristics have predominantly been associated with misdiagnosis, inappropriate treatment^16^ and (notably for forensic services) high-impact failures of case management that have resulted in fatalities.^17^ Heuristics are predominantly presented as a source of error; there is a widespread assumption that cognitive short-cuts are necessarily bad, but also a growing realisation that the efficacy of heuristics is open to debate.^18^ A counter argument has therefore arisen against the *a priori* assumption that heuristics are necessarily bad. This counter argument is that heuristics may have many practical benefits that counterintuitively can include accuracy, transparency and efficiency.^19^ Furthermore, it has been argued that heuristics in practice-based medicine drive the iterative refinement of decision-making and allow clinicians to learn to focus on outcome-relevant features through reflection on the consequences of clinical decisions that they observe directly over time.^20^ The possibility that heuristics may have some value is particularly interesting to forensic practitioners who have become accustomed to the notion that clinical opinion is less reliable than actuarial or structured clinical judgement tools.^21^ The nature and extent of the use of heuristics in forensic practice, however, remain largely unknown, despite the substantial potential benefits and costs for these forms of decision-making.

Until recently, gatekeeping decisions have relied on clinical experience along with local definitions of security need. Structured clinical judgement tools with robust psychometric properties are now beginning to influence gatekeeping practice and may be preferred because they promise increased transparency and uniformity in decision-making. Despite widespread knowledge of the DUNDRUM quartet^22^ among secure service personnel, the extent to which this tool has been adopted and implemented in gatekeeping decision-making is unknown and has only recently become a subject for evaluative investigation.^23^

Kennedy^24^ lists violence, immediacy of risk, specialist forensic need, absconding behaviour and public confidence as the assessment guidelines that should determine security placement of patients. The Dangerousness, Understanding, Recovery and Urgency Manual^22^ was developed as an aid to clinical decision-making, and includes a scale designed to assist psychiatric triage (DUNDRUM-1). The DUNDRUM-1 is an 11-item risk assessment tool that uses both scores on individual items and the distribution of those scores (a case should score mostly in one category) to inform judgement. The tool is reported to have excellent predictive validity, high internal consistency and good interrater reliability.^25^ The DUNDRUM-1, therefore, has potential for assisting clinical decision-making regarding patient placement and for evaluating the correspondence between patient characteristics and secure service placement. The DUNDRUM-1 also has the potential to measure the influence of heuristic bias in clinical decision-making.^26^^,^^27^ The DUNDRUM quartet is a third-generation risk assessment tool, with items that are anchored in specific definitions of low, medium and high security that do not take account of the contextual factors within individual secure units (patient acuity, patient dynamics, workforce skill, philosophies of care and so on). Nevertheless, the DUNDRUM allows the initial quantification of the specific nature of anchoring that may underpin clinical decision-making in relation to allocating people to levels of security.

## Aims and hypotheses

First, the current study was designed to extend examination of the external validity of the DUNDRUM-1 psychiatric triage tool to a sample that was different from the original validation sample,^22^^,^^25^ and also different from the first external demonstration^23^ of the DUNDRUM-1 as a useful support for clinical decision-making. Significant alignment between DUNDRUM-1 scores and patient placement would confirm the external validity of the tool. The second objective of the current study was to use the DUNDRUM-1 as a ‘best practice’ framework in which to examine anchoring heuristics in secure service gatekeeping decisions. Anchoring would be apparent if, following regression, one (or a narrow set) of the DUNDRUM-1 items were able to explain a disproportionately large fraction of variance in patient placement.

## Method

### Sampling

The study was approved by the research ethics committee of Cardiff Metropolitan University's School of Health Sciences, and as a service evaluation by the relevant local National Health Service (NHS) Health Board Research and Development department. All patient characteristics were extracted from written narrative reports that had been triggered by a referral requesting admission to secure services. In every instance, permission was sought from the original report author; no requests were declined.

Characteristics of the sample were extracted from the All Wales Secure Services database of immediate post ‘referral-to-service’ (gatekeeping) assessment reports between January 2010 and June 2013. This database is used in the management of Welsh secure service patient placements. Cases were selected if they had not previously been referred either for treatment or assessment by secure services. First-time referral was used as an inclusion criterion to control for possible confounding of assessment recommendations by previous placement of patients. The database was interrogated sequentially for cases where a psychiatric report was available and the destination of the patient recorded. A total of 121 assessment reports were present in the database for the sampling period.

Of the 121 cases, 50 patients were admitted to medium security, 49 were admitted to low security and 22 remained in open conditions. Reports included comprehensive details of the index offence, forensic history, Mental Health Act 1983 status, psychiatric history, diagnoses, previous non-criminalised violence and/or self-harm, and the gatekeeping recommendation for security level.

### Design

The study used a between-subjects retrospective cohort design.^28^ All cases involved first-time referrals for secure care to avoid biasing of placement decisions by previous secure referrals. Neither of the raters had any involvement in the referral process for any of the patients included in the current study. Both were also blind to the referral outcomes at the time of completing the assessments.

### Materials

The Dangerousness, Understanding, Recovery and Urgency Model^22^ was used as the method for rating patient characteristics at the time of first referral. DUNDRUM-1 is the triage component of the DUNDRUM Quartet and consists of 11 items that rate patient triage features on a 0–4 scale, where 0 is very low severity and 4 is high severity. Kennedy *et al*.^22^ suggest that people who mostly score 4 (across the range of the items on the scale) will initially require conditions of high therapeutic security (i.e. Special Hospital). A person who mostly scores 3 is likely to need conditions of medium security, and a person who is mostly rated 2 will be best placed in conditions of psychiatric intensive care (acute low security). A patient rated as 1 on most of the items should be safely treated in an open setting, and a person mostly rated 0 may be cared for in a community setting.

The DUNDRUM-1 was selected on the basis of its psychometric properties. The DUNDRUM-1 has been reported to have acceptable validity and reliability.^25^ For instance, the scale has been reported to have good internal consistency (Cronbach's α: 0.95) and also good interrater reliability; the kappa statistic could be rated for seven of the 11 items and was greater than 0.85 for each of these.

### Procedure

The DUNDRUM-1 was applied to each report by one of 2 raters (D.L. & T.D.). Both raters had successfully completed reliability workshops for the Historical Clinical Risk Management-20 (HCR-20), Sexual Violence Risk-20 (SVR-20) and Psychopathy Checklist – revised (PCL-R); they had achieved high interrater reliability with HCR-20 assessment (kappa for both: 0.9) and were therefore assumed to be reliable in the application of other similar structured clinical judgement tools (DUNDRUM-1). For each case, each item of the DUNDRUM-1 was scored by either D.L. or T.D. in accordance with item score definitions published for DUNDRUM-1.^22^

### Methods of analysis

All statistical analyses were conducted using SPSS version 22 (IBM). The alpha criterion was set throughout at 0.05, and alpha was adjusted for multiple comparisons using the Holm–Bonferroni method.^29^

The index of predictive validity reported here is the area under the curve (AUC). In both the current study and the comparison data^23^ the AUC was used to determine the ability of the individual DUNDRUM-1 item scores (and total score) to discriminate between cases admitted to security (low or medium secure) versus those who were not admitted to security (open conditions). AUC values can range between 0 and 1 (0 = perfect negative prediction, 0.5 = no predictive validity and 1 = perfect positive prediction). Higher AUC values indicate increased predictive validity, 0.5 acts a reference and 95% confidence intervals are used to determine whether predictive validity is superior to chance; where the lower bounds of the 95% CI were below 0.5, the null hypothesis (predictive validity is no better than chance) was accepted. Similarly, where confidence intervals overlapped, there were no significant differences in predictive validity between DUNDRUM-1 items or samples.

The next set of analyses was designed to determine whether scores for DUNDRUM-1 items differed between patients allocated to each of the three tiers of security (open conditions, low security or medium security) in the current sample. Total DUNDRUM-1 scores and individual DUNDRUM-1 item scores for these three groups were compared using Kruskal–Wallis analyses, *post hoc* contrasts between specific pairs of groups used Mann–Whitney U, and alpha inflation was controlled using the Holm–Bonferroni^29^ method.

The final set of analyses used multiple ordinal regression to explore the extent to which DUNDRUM-1 items might individually, or in combination, explain patient placement. Analysis used multiple ordinal regression because of the ordinal nature of the independent (DUNDRUM-1) and dependent (patient placement) variables. Models were designed using the enter method in order to explore possible structures in decision processes.

## Results

**Fig. 1:**
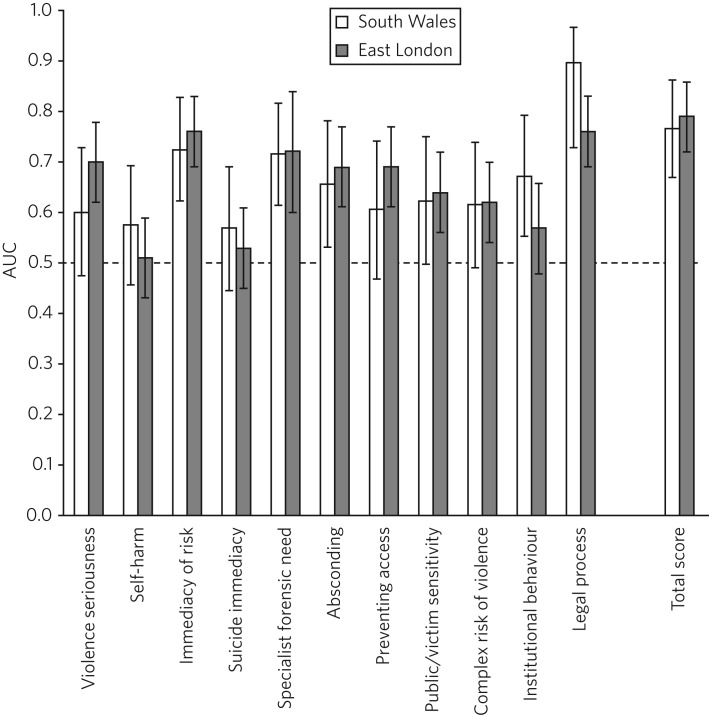
Comparison of AUC for DUNDRUM-1 total and item scores between East London (Freestone *et al*.^23^) and South Wales (current sample). AUCs reflect the validity of DUNDRUM-1 items for discriminating individuals admitted to secure services (low or medium secure) from those not admitted to secure services. Error bars indicate 95% confidence intervals. The hatched reference line at AUC = 0.5 indicates the line of no information.

For comparison, the results of the AUC analyses for the current sample and for the Freestone *et al*.^23^ sample are presented in Fig. 1. Predictive validities of total DUNDRUM-1 scores for both samples were superior to chance, and the AUC values and confidence intervals were remarkably similar between the two samples. The picture for individual DUNDRUM-1 items was more complex and not wholly consistent between the two samples. The two samples yielded similar significant AUC results for the following five items: immediacy of risk, specialist forensic need, absconding and legal process. The two samples were also consistent in finding that neither self-harm nor suicide immediacy predicted secure admissions. The two samples yielded inconsistent AUC results for violence seriousness, preventing access, public/victim sensitivity, complex risk of violence and institutional behaviour. Violence seriousness predicted secure placement in the East London sample but not in the South Wales sample; the same pattern was observed for preventing access, public/victim sensitivity and complex risk of violence. The opposite pattern was apparent for the institutional behaviour item; this item showed significant predictive validity for the South Wales sample but not for the East London sample. The preceding analysis served as a means of assessing the predictive validity of the DUNDRUM-1 for discriminating between patients admitted to secure services versus those not admitted to secure services. The next series of analyses were concerned with whether either DUNDRUM-1 total scores or individual DUNDRUM-1 items differed significantly between three groups of patients in South Wales.

### External validity, total DUNDRUM-1 scores

**Fig. 2:**
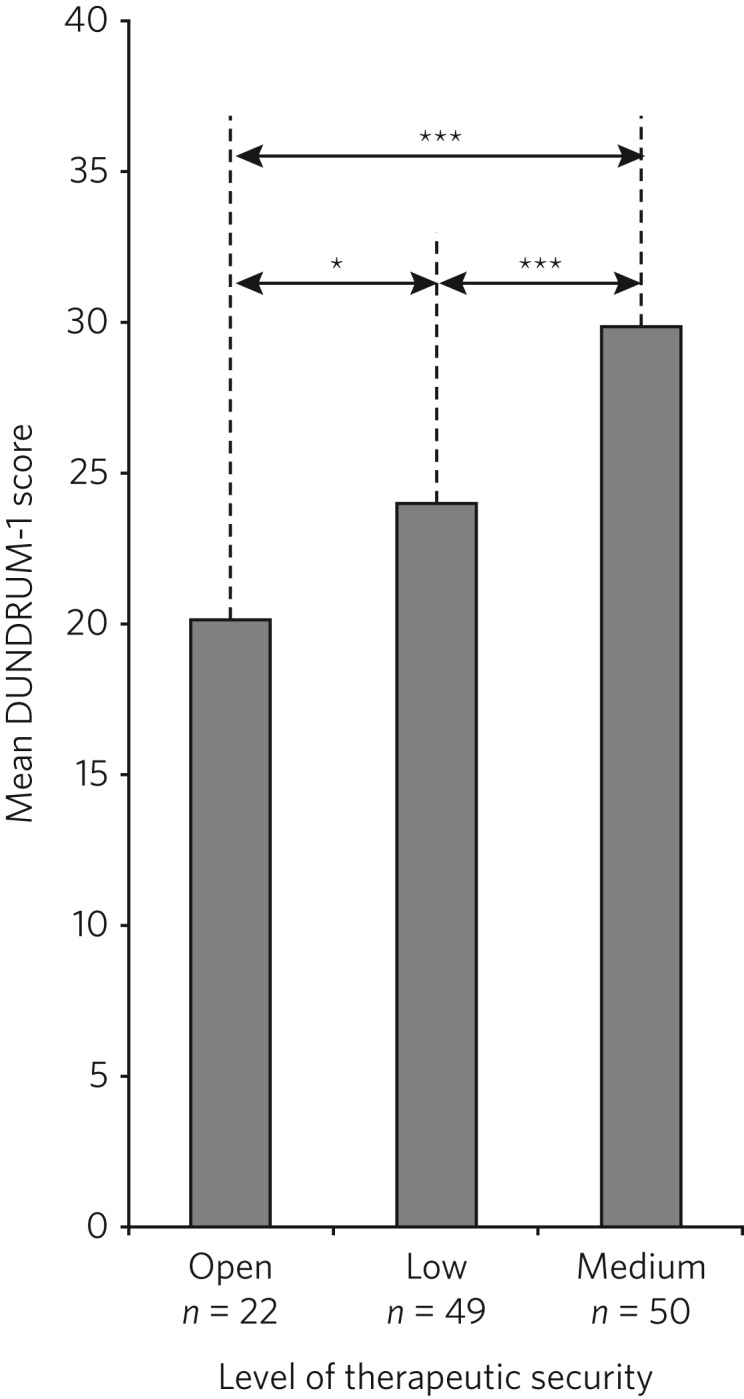
Mean DUNDRUM-1 scores for the patients allocated to open conditions, low and medium security. Error bars show the standard error of the mean (*P < 0.050; ***P < 0.001).

Fig. 2 presents the mean total DUNDRUM-1 scores separately for the patients who stayed in open conditions and for those who were admitted to either low or medium secure care. The mean scores suggest appropriate alignment between DUNDRUM-1 criteria and clinical gatekeeping decisions; mean scores increased as the levels of therapeutic security increased. This impression of the total DUNDRUM-1 scores was confirmed by the association between patient placement and total DUNDRUM-1 scores, *H* (*n* = 121, d.f. = 2) = 20.737, *P* < 0.000. Furthermore, *post hoc* comparisons with Mann–Whitney and alpha adjustment revealed that mean total DUNDRUM-1 scores for each of the groups differed from the other two groups (open *v.* low, mean difference = 3.86, *P* < 0.050; low *v.* medium, mean difference = 5.78, *P* < 0.005; and open *v.* medium, mean difference = 9.64, *P* < 0.005).

### External validity, individual DUNDRUM-1 items

**Fig. 3:**
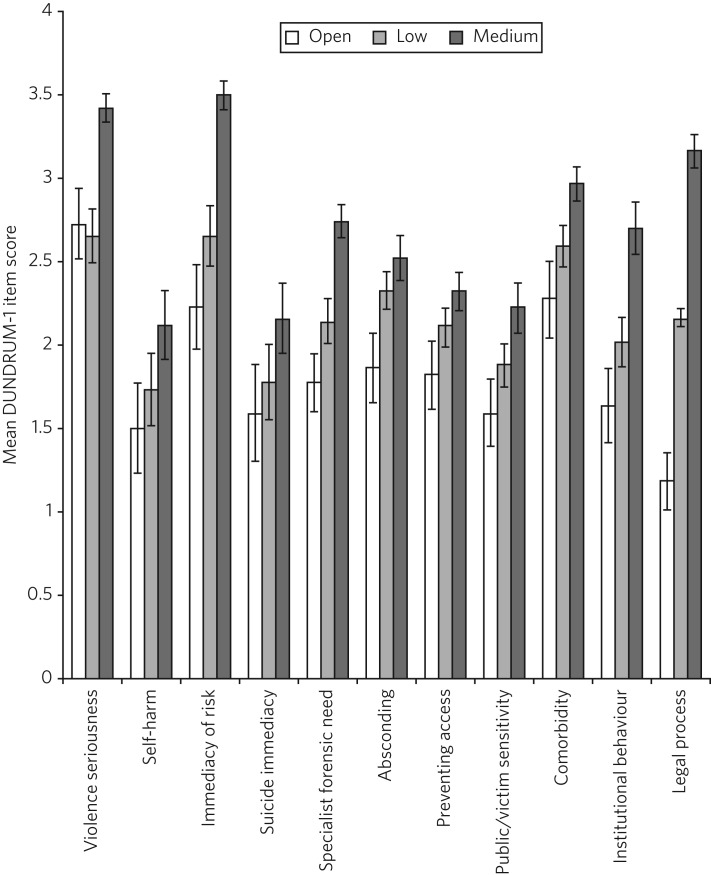
Mean DUNDRUM-1 item scores for patients who stayed in open conditions, and those who were admitted to either low or medium security. Error bars show the standard error of the mean.

Fig. 3 presents the mean scores for individual DUNDRUM-1 items for three groups of patients referred to the South Wales Forensic Mental Health Service: those who subsequently remained in open conditions, those admitted to low security and those admitted to medium security. Between-group differences and successive increases in the mean scores for individual items indicated that patient placement had been consistent with DUNDRUM-1 criteria. Inspection of the data in Fig. 3 suggested that patient placement was not consistently aligned with all of the DUNDRUM-1 items, for example, there was clear separation in mean scores between the groups for the legal process item but poor separation for the self-harm seriousness item. In order to explore this impression of the association between items and patient placement, a series of Kruskal–Wallis tests were conducted, one for each of the 11 items of the DUNDRUM-1.

The results of the multiple Kruskal–Wallis analyses are presented in Table 1. The impression that some DUNDRUM-1 criteria fitted service placement more accurately than others was confirmed. Only seriousness of violence, immediacy of risk, specialist forensic need, legal process and institutional behaviour were significantly associated with patient placement. Placement was not associated with absconding risk, complex violent need, victim sensitivity/public confidence, preventing access, self-harm seriousness or immediacy of suicide risk. It is worth noting, however, that several items (absconding risk, complex violent need and victim sensitivity/public confidence), if tested individually, would have been regarded as statistically significant but were rejected here after alpha adjustment.

**Table 1:** Kruskal–Wallis analysis of individual DUNDRUM-1 items

DUNDRUM-1 item	Observed *P*-value	Adjusted alpha	Significance
Legal process	<0.000	0.005	***
Immediacy of risk	<0.000	0.005	***
Specialist forensic need	<0.000	0.005	***
Seriousness of violence	<0.000	0.006	***
Institutional behaviour	<0.001	0.007	**
Absconding risk	<0.050	0.008	ns
Complex violent need	<0.050	0.010	ns
Victim sensitivity/public confidence	<0.050	0.013	ns
Preventing access	>0.050	0.017	ns
Self-harm seriousness	>0.050	0.025	ns
Immediacy of suicide risk	>0.050	0.050	ns

Alpha was adjusted for multiple comparisons using the Holm–Bonferroni method. DUNDRUM-1 items were sorted on the basis of observed *P*-value (reading down, lowest to highest). ***P < 0.000; **P < 0.001; ns, not significant.

The above Kruskal–Wallis analysis was used to select predictor variables for regression modelling of gatekeeper decision-making and identified five significant potential predictor variables. The current sample of 121 cases with five predictor variables therefore exceeded the normal power convention (30) in standard regression analyses (104 + number of predictors = 109).

### Analysis of heuristics

With an ordinal dependent variable, an ordinal regression was chosen. The distribution of the dependent variable was skewed towards the higher security levels (see Fig. 2), so the complementary log-log function was used.

The predictors for the model were those highlighted in Table 1 by the Kruskal–Wallis analysis (seriousness of violence, immediacy of risk, specialist forensic needs, legal process and institutional behaviour). The model was a significant predictor of level of security assignment (χ^2^ = 236.6, d.f. = 5, *P* < 0.001), with estimates of variance accounted for ranging between 86% (Cox and Snell) and 98% (Nagelkerke). The goodness of fit tests (Pearson and deviance) were both non-significant (*P* = 1.000 for both). The test of parallel lines was also non-significant (χ^2^ = 0.0, d.f. = 5, *P* = 1.000). Parameter estimates are shown in Table 2. The two significant predictors in this initial model were legal process and immediacy of risk.

**Table 2:** Parameter estimates of variables predicting assigned level of security (whole model)

		Estimate	s.e.	Wald	d.f.	*P*-value
Predictors	Seriousness of violence	−0.06	0.19	0.11	1	>0.050
	Immediacy of risk	0.36	0.17	4.70	1	**<0.050**
	Specialist forensic needs	0.24	0.20	1.47	1	>0.050
	Legal process	1.81	0.28	42.22	1	**<0.001**
	Institutional behaviour	0.22	0.15	0.02	1	>0.050

Significant *P* values are highlighted in bold.

To examine the variance accounted for by different elements of the model, the analysis was repeated with only legal process and immediacy of risk (the elements that were significant in the initial model). Legal processes and immediacy of risk were significantly associated (Kendall's tau = 0.255, *P* < 0.010). The simpler model was a significant predictor of security assignment (χ^2^ = 166.4, d.f. = 2, *P* < 0.001); the estimates of variance accounted for were lower than when all five predictors were included in the model, between 75% (Cox and Snell) and 85% (Nagelkerke). The goodness of fit tests were both non-significant (Pearson *P* = 0.940, deviance *P* = .530), see Table 3.

**Table 3:** Parameter estimates of variables predicting assignment to level of security

		Estimate	s.e.	Wald	d.f.	*P*-value
Predictors	Immediacy of risk	0.35	0.13	7.83	1	**<0.005**
	Legal process	1.56	0.25	40.73	1	**<0.001**

Significant *P*-values are highlighted in bold.

The remaining three predictors (seriousness of violence, special forensic needs and institutional behaviour) were then considered without legal Process and immediacy of Risk, see Table 4. The resulting model was a significant predictor of security assignment (χ^2^ = 34.9, d.f. = 3, *P* < 0.001); estimates of variance accounted for ranged between 14% (McFadden) and 28% (Nagelkerke). The goodness of fit tests were both non-significant (Pearson *P* = 0.200, deviance *P* = 0.080). Specialist forensic need was correlated with both institutional behaviour (Kendall's tau = 0.342, *P* < 0.001) and seriousness of violence (Kendall's tau = 0.258, *P* < 0.005); however, institutional behaviour scores were independent of seriousness of violence (Kendall's tau = 0.025, *P* > 0.050).

**Table 4:** Parameter estimates of variables predicting assigned level of security (initially excluded predictors)

		Estimate	s.e.	Wald	d.f.	*P*-value
Predictors	Seriousness of violence	0.37	0.16	5.01	1	**<0.050**
	Specialist forensic needs	0.30	0.13	5.26	1	**<0.050**
	Institutional behaviour	0.33	0.13	6.26	1	**<0.050**

Significant *P*-values are highlighted in bold.

## Discussion

### External validity of the DUNDRUM-1 triage tool

The weaknesses of unsupported clinical risk assessment have been the subject of debate for more than 30 years, and a growing number of tools have been designed with the intent of maximising consistency and transparency; however, the application of violence risk assessment tools has been extensively criticised.^30^ The DUNDRUM quartet^22^ has been designed specifically as a support tool for clinical judgements at important junctures in patient care (admission to hospital, increasing/decreasing therapeutic security and discharge). One potential weakness of the DUNDRUM quartet is that it was developed in a specific service context, and its practical relevance depends heavily upon its generality. Currently, the validity of this tool rests upon work conducted predominantly by its authors; very little is known about how well the contents of the DUNDRUM quartet generalise to decision-making in other services.

Overall, the findings of the current study support the predictive and external validity of the DUNDRUM-1 triage tool as a clinical decision aid for first-time referrals to secure psychiatric services. Total scores on the DUNDRUM-1 differed between all three of our groups of patients (open conditions, low secure and medium secure), and scores increased as levels of security allocation increased. The main measure of predictive validity was the AUC, and our findings, along with those of Freestone *et al*.,^23^ indicate acceptable discriminative power for the DUNDRUM-1; total scores on the DUNDRUM-1 yielded AUC values between 0.75 and 0.8, with lower bounds for 95% CIs that were comfortably above chance. It is notable, however, that these AUC values were lower than that reported earlier (AUC = 0.984) when the DUNRUM-1 was used to discriminate between court cases in which individuals were admitted or not admitted to psychiatric care.^25^ By contrast, all of the individuals in the samples reported by Freestone *et al*.^23^ and here were referred to services, presumably because their offending and/or clinical presentation caused sufficient concern to prompt forensic assessment. It is therefore unsurprising that our AUC values were lower than those reported earlier, because any sample referred to specialist services will have been relatively homogenous compared with a ‘randomly’ selected and clinically heterogeneous court sample. This finding shows that the predictive validity of the DUNDRUM-1 depends to some extent upon the context in which it is being applied, and its external validity may therefore be constrained.

The pattern of findings for the predictive validity of individual DUNDRUM-1 items was not simple. First, both the current study and Freestone *et al*.^23^ found that self-harm and suicide risk were not related to decisions following referral for possible admission to secure conditions. This finding suggests that secure admissions in South Wales and East London were not influenced by the severity of the risk that individuals posed to themselves. It is plausible that differences in service configuration (e.g. the degree of integration between secure and general services) between the UK and Eire could explain the restricted external validity of these two items. Alternatively, this could reflect a difference in emphasis on public protection between services in the UK and in Eire. Further research would have to be conducted in order to determine the source of this important difference.

As well as the differences between the two UK samples and the original validation sample for the DUNDRUM-1, there were also a number of differences between the two UK samples that may indicate local constraints on the validity of some of the tool's items. There was agreement in AUC analysis between the South Wales and East London samples for six of the 11 DUNDRUM-1 items (including non-significant results for suicide and self-harm). AUC results therefore differed for five items, including seriousness of violence, preventing access, public/victim sensitivity, complex risk of violence and institutional behaviour. Therefore, the influence of these factors on secure admissions decision-making differed between South Wales and East London.

Overall, the above interpretation of the external validity of the DUNDRUM-1 triage tool raises some difficult questions. Are generic clinical judgement tools viable for applications across contexts, where there appear to be so many differences in emphasis between services and in the predictive accuracy of the tool? Might locally defined clinical judgement tools provide an alternative approach that would be more contextually relevant, especially in light of increasing devolution of health service configuration and governance between regions of the UK?

### Anchoring heuristics in clinical decision-making

The emphasis in the forensic literature has been on the possible contribution of heuristic decision-making to preventable fatalities; however, only a minority of such events are in fact predictable, even when heuristics could have been minimised by the application of risk assessment tools.^31^ The current work makes a novel contribution, because it focused instead on characterising the nature of heuristics applied by gatekeepers when patients were allocated to different levels of therapeutic security at the point of first referral to secure services.

The information for rating all of the items of the DUNDRUM-1 was readily available in all of the patient referral reports that were analysed in the current study. The gatekeeping clinicians were therefore clearly collating and reporting patient characteristics relevant to all 11 of this tool's items, even though the gatekeepers were not using the DUNDRUM-1 in their decision-making. The DUNDRUM triage tool, therefore, showed excellent face validity with respect to the information gathering practices in secure triage assessment. In the following, we consider whether all of this information actually informed clinical triage decisions to the same extent, or whether gatekeeping decisions instead reflected the operation of heuristic bias.

**Fig. 4:**
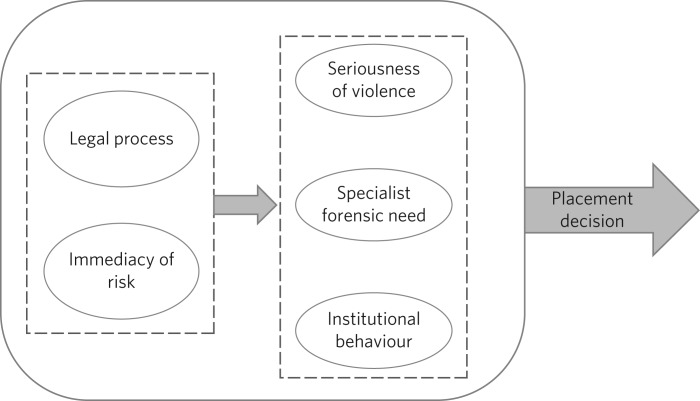
Legal process and immediacy of risk served as heuristic anchors that may have acted as a heuristic frame for secondary consideration of seriousness of violence, specialist forensic need and institutional behaviour.

The second objective of the current study was to use regression analyses of individual DUNDRUM-1 items and gatekeeping decisions to examine the nature of heuristics in the decisions made by clinicians in South Wales. Our analyses revealed the presence of anchoring^12^ that was biased in favour of a subset of DUNDRUM-1 items. Two of the 11 (legal process and immediacy of risk due to mental disorder) explained a disproportionately large fraction (between 86% and 98%) of the variance in patient placement. When these two potent predictors were removed from the model, three further variables (seriousness of violence, specialist forensic need and institutional behaviour) were shown to predict security level but explained a more modest proportion (between 14% and 28%) of variance in decision-making. Taken together, the two regression analyses suggest a two-tiered, hierarchical heuristic (see Fig. 4) that was primarily anchored by two items, legal process (which reflected the least restrictive option acceptable to all parties) and immediacy of risk due to mental disorder; the secondary tier included consideration of seriousness of violence, specialist forensic need and institutional behaviour.

Our heuristic analysis rested on the assumption that each of the items of the DUNDRUM-1 tool should influence decision-making to the same extent. This is a common assumption in the construction of risk assessment tools (PCL-R; HCR-20), but it often does not reflect the subjective weighting given to these characteristics by clinicians during assessments. Further application of regression methods with the DUNDRUM-1 and other assessment tools could further elaborate the extent and nature of anchoring heuristics in many aspects of clinical decision-making regarding people with mental illness.

### Limitations

Anchoring and framing heuristics are closely interrelated,^11^ and it is plausible that consideration of two or more of the patient characteristics may have interacted: consideration of some items may have acted as frames for subsequently considered items. Unfortunately, the design of the current study did not allow more detailed exploration of such interactive cognitive processes; the contribution of framing heuristics here must remain unknown.

Examination of the referral reports yielded information on a great many factors that did not form part of the items included in the DUNDRUM-1. Gatekeepers were therefore routinely collecting and assessing information beyond the scope of the DUNDRUM-1. Our analysis depended on applying the DUNDRUM-1 as a best practice framework, and it could not capture the possible contribution of these other factors to triage assessment decisions. We conclude that the heuristic in triage decisions was anchored on only two factors. It is possible that selection of the DUNDRUM-1 as the best practice framework for the current analysis may have artificially constrained the resulting heuristic and clinicians may have been basing their decisions on a wider set of factors.

The current study does not inform about any relationships between triage decisions and either health or forensic outcomes. Individual progress monitoring and aggregated data on outcomes for patients placed in secure mental health care (cf. Centre for Mental Health, 2011) remains underdeveloped in the UK.

## About the authors

**Daniel Lawrence** is a Forensic Psychologist, working at various locations in Wales for the Priory Group, UK. **Tracey-Lee Davies** is currently acting Head of Nursing for Child and Adolescent Mental Health Services (CAMHS) in Bridgend, UK. **Dr Ruth Bagshaw** is the Lead Consultant Clinical and Forensic Psychologist in Specialist Services for ABMU Health Board, Wales. **Dr Paul Hewlett** is Senior Lecturer in Applied Psychology, Cardiff Metropolitan University, UK. Professor Pamela Taylor holds the Chair in Psychiatry at the Division of Psychological Medicine and Clinical Neurosciences, Cardiff University, UK. **Dr Andrew Watt** is a Reader in Applied Psychology at the Department of Applied Psychology, Cardiff Metropolitan University, UK.
